# Research on the Relationship between Resistivity and Resistance between Two Points on RCS Test Model

**DOI:** 10.3390/s23031139

**Published:** 2023-01-19

**Authors:** Yacong Wu, Jun Huang, Lei Song

**Affiliations:** School of Aeronautic Science and Engineering, Beihang University, Beijing 100191, China

**Keywords:** four-probe method, RCS test model, surface resistance, sheet resistance, two-probe method

## Abstract

Surface conductivity is one of the key factors in judging whether the RCS (Radar Cross Section) test model is qualified, but the accuracy of traditional detection methods is insufficient. Furthermore, the resistance between two points obtained by traditional methods cannot be directly applied to the electromagnetic simulation analysis of the test model. In this paper, the theoretical model of the relationship between resistivity and resistance between two points on the model surface is proposed. The simulation method for the resistance between two points on the model surface is established. The advantage of the method proposed in this paper compared with the traditional method in detecting the surface resistance of the model is demonstrated intuitively. The experiments are carried out on ITO (Indium Tin Oxide) conductive films with several dimensions and resistivity. Results show that the measured resistance between two points on the model surface is highly consistent with the theoretical and simulated values. Moreover, the comparison of experiments shows that the measurement error of the traditional method is 150% to 200% higher than that of the method proposed in this paper.

## 1. Introduction

With the development of processing technology, non-metallic materials, such as plastic, resin, and foam, are widely used in industrial manufacturing due to their utility, weight, high precision, and low cost [1,2]. Since the electromagnetic properties of non-metallic materials are quite different from those of metal materials, it may be necessary to metalize the surface of non-metallic components in certain application scenarios that require conductivity [3]. In order to ensure that the components can achieve the requisite electromagnetic characteristics after surface metallization, surface conductivity is typically used as the control index of metallization process.

In general, to assure test accuracy while conducting scaled RCS test on a full-size metal model, the actual test model must meet the ideal conductor assumption [4]. It is easy to achieve this requirement by processing the test model with metal. However, compared with sheet metal processing, emerging 3D printing has more significant advantages in terms of prototyping, such as low cost, short production cycle and lightweight. Typically, the test model is constructed using 3D printing resin materials [5,6]. Due to the skin effect of the current in the ideal conductor [7], the electromagnetic scattering characteristics similar to the metal model can be achieved by spraying a metal layer with sufficient conductivity on the surface of the 3D printing model [8]. As the performance index of conductive coating, the current Chinese military standard (GJB5022-2001) [9] requires that the resistance measured at intervals of 300 mm between two points on the surface of the RCS test model should be less than 1 ohm.

At present, an ordinary ohmmeter with two probes is commonly used in the RCS test to measure the resistance between two points on the model surface to judge whether the conductivity of the model surface meets GJB5022-2001. However, according to Heaney [10] and Long [11], the contact resistance will have a more significant impact on measurement values when two-probe method is used to measure the resistance on the model surface. The use of two-probe method in the RCS test will erroneously judge the conductivity of model surface.

In addition, although the parameter directly measured and stipulated by the Chinese military standard is the resistance between two points on the model surface, the parameter used in the electromagnetic simulation is the resistivity of the model surface [12]. Since the relationship between the two values is not established and cannot be converted, the model with conductive paint sprayed on the surface cannot be substituted into the simulation. In most cases, only the RCS test may be used to determine the difference between the conductive film coating model and the metal model [13].

In summary, if a more precise evaluation of whether the conductive coating meets the requirements is required, or if simulation analysis on the conductive coating model is desired, there are two problems that must be resolved immediately:

1. Propose an accurate method for measuring the resistance between two points on the model surface.
2. Establish the relationship between the resistivity and resistance between two points at any interval on the model surface.

The purpose of this paper is to propose a theoretical model of the relationship between resistivity and resistance between two points on the model surface, establish a simulation method for the resistance between two points on the model surface, and verify the theoretical model and simulation method through experiments. Finally, a more accurate method for evaluating the surface conductivity of the RCS test model is developed.

## 2. Theorical Model of Relationship between the Resistivity and Surface Resistance

For the general 3D processing RCS test model, the conductivity is entirely provided by the conductive paint on the surface. The internal insulating structure has no effect on the RCS test for the reason of wave-transmissive [14]. The conductive paint on the outer layer can be regarded as a conductive film since the thickness is small enough relative to the size [15]. Therefore, the following sections will directly use conductive film as the research object.

**Figure 1:**
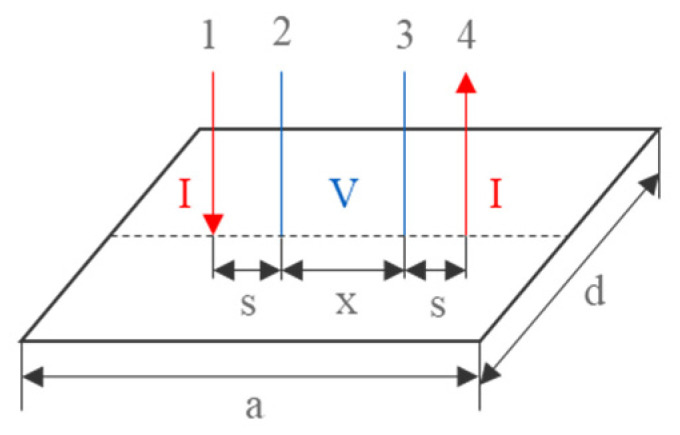
Arrangement of a four probe on a rectangular conductive film.

Consider a rectangular conductive film sample with dimensions a and d. Place four points on it and the distance between points 1, 2 and points 3, 4 is fixed, both are s. The distance between points 2, 3 is variable, set to x, as is shown in Figure 1. The labels 1, 2, 3, 4 in the figure represent four probes, “I” represents current and “V” represents voltage.

When using the four-probe method to measure the resistance between two points of a finite-size conductive film, the current flows in through probe 1 and flows out from probe 4, which is equivalent to arranging a positive current source in point 1 and a negative current source in point 4. The resistance between points 2 and 3 can be obtained by solving the potential between those two points. That is, the essence of the problem is to solve the contribution of the current sources at points 1 and 4 to the potential of points 2 and 3.

**Figure 2:**
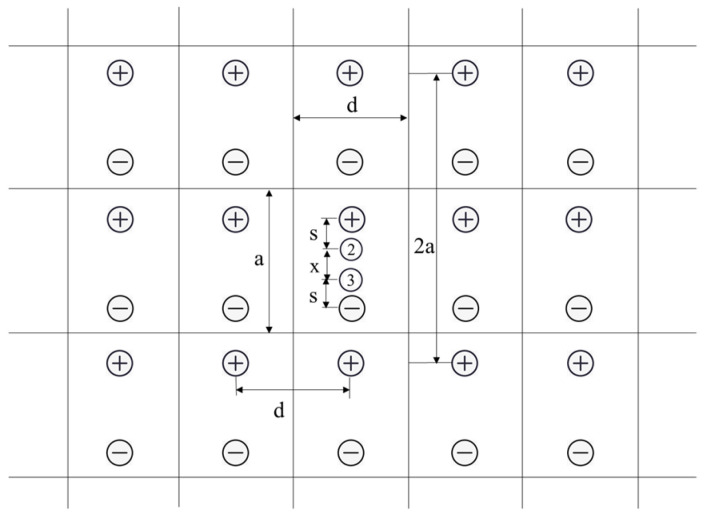
System of images.

To obtain the voltage between points 2 and 3, an infinite arrangement of dipoles must be considered [16], as shown in Figure 2. All contribute to the voltage between points 2 and 3.

**Figure 3:**
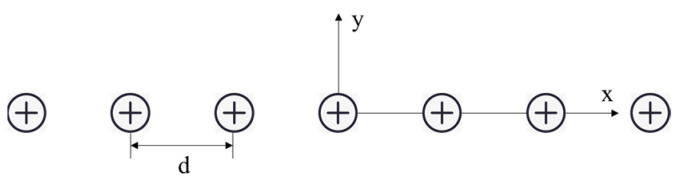
Coordinate system for a linear arrangement of current sources.

F. Ollendorff [17] gives the potential distribution for an infinite number of current sources, arranged in a line and equally spaced. With a coordinate system as in Figure 3, the potential is

(1) $$\varphi -\varphi_{0}=-\frac{IR_{s}}{2\pi }\ln2\sqrt{\sin^{2}\frac{\pi }{d}x+\sinh^{2}\frac{\pi }{d}y}$$

where I is the current, $R_{s}$ stands for sheet resistance, which is a physical quantity used to characterize the resistivity of film and can be expressed as the ratio of material resistivity of the film to thickness.

By (1), the problem of solving the potential between points 2 and 3 can be converted to solving the superposition of potential caused by the infinite lines of current sources at points 2 and 3. Since the current source extends infinitely in the x-direction, for each row of current sources, the x-coordinates of points 2 and 3 are both zero, and the (1) can be simplified as

(2) $$\varphi -\varphi_{0}=-\frac{IR_{s}}{2\pi }\ln(e^{\pi \frac{y}{d}}-e^{-\pi \frac{y}{d}})$$

Each row of sources thus contributes to the potential between points 2 and 3 the amount

(3) $$\Delta \varphi_{n}=-\frac{IR_{s}}{2\pi }\ln(\frac{e^{\pi (y_{n}+x)/d}-e^{-\pi (y_{n}+x)/d}}{e^{\pi y_{n}/d}-e^{-\pi y_{n}/d}})$$

where $y_{n}$ is the distance from point 2 to the center source in line n.

Considering symmetry, only the current sources in the lower half plane are to be considered in the following derivation and the result must be multiplied by 2.

Introduce mathematical simplification:

(4) $$-\ln(e^{\pi y/d}-e^{-\pi y/d})=-\ln e^{\pi y/d}(1-e^{-2\pi y/d})=-\pi \frac{y}{d}-\ln(1-e^{-2\pi y/d})$$

Equation (3) can be rewritten as:

(5) $$\begin{array}{cc} \frac{2\pi }{IR_{s}}\Delta \varphi_{n} & =\pm [-\pi \frac{y_{n}+x}{d}-\ln(1-e^{-2\pi (y_{n}+x)/d})+\pi \frac{y_{n}}{d}+\ln(1-e^{-2\pi y_{n}/d})]\\ & =\pm [-\pi \frac{x}{d}-\ln(1-e^{-2\pi (y_{n}+x)/d})+\ln(1-e^{-2\pi y_{n}/d})] \end{array}$$

with + standing for a positive source and − for a negative source.

Normalize the length variable, let:

$$d=\frac{d}{s},\;a=\frac{a}{s},\;y=\frac{y}{s},\;x=\frac{x}{s},\;s=1$$

To sum the ln terms the logarithm can be expanded:

(6) $$-\ln(1-x)=x+\frac{1}{2}x^{2}+\frac{1}{3}x^{3}+\cdots$$

The two items related to n in (5) can be expressed as

(7) $$\pm \sum_{m=1}^{\infty }\frac{1}{m}[e^{-2\pi (y_{n}+x)m/d}-e^{-2\pi y_{n}m/d}]$$

where $y_{n}=A_{i}+n\cdot 2a,(i=1,2,3,4)$, the case y=1 is treated separately and not included in $y_{n}$.

For + source:

$$A_{2}=a+1\;A_{4}=2a-x-1$$

For − source:

(8) $$A_{1}=2a+1\;A_{3}=a-x-1$$

Considering the superimposed value of the current source contribution of each row, for each m in (7), let n perform series superposition in the interval of [0,∞):

(9) $$\begin{array}{cc} a_{m} & =\sum_{i=1}^{4}\sum_{n=0}^{\infty }\pm \frac{1}{m}[e^{-2\pi (A_{i}+n2a+x)m/d}-e^{-2\pi (A_{i}+n2a)m/d}]\\ & =\sum_{i=1}^{4}\sum_{n=0}^{\infty }\pm \frac{1}{m}[e^{-2\pi A_{i}m/d}\cdot e^{-4\pi nam/d}\cdot [e^{-2\pi xm/d}-1]] \end{array}$$

It can be seen from (9) that only one item in $a_{m}$ is related to n, and this is a geometrical series in n. As is known:

(10) $$\sum_{n=0}^{\infty }x^{n}=\frac{1}{1-x}$$

Summing the items with respect to n in $a_{m}$, (9) can be written as:

(11) $$a_{m}=\sum_{i=1}^{4}\pm \frac{1}{m}[\frac{\left(e^{-2\pi xm/d}-1\right)}{\left(1-e^{-4\pi am/d}\right)}\cdot e^{-2\pi A_{i}m/d}]$$

Substituting $y_{n}=A_{i}+n\cdot 2a,(i=1,2,3,4)$ into (11), we obtain:

(12) $$\begin{array}{cc} a_{m} & =\frac{1}{m}\cdot \frac{\left(e^{-2\pi xm/d}-1\right)}{\left(1-e^{-4\pi am/d}\right)}[e^{-2\pi (a+1)m/d}+e^{-2\pi (2a-x-1)m/d}-e^{-2\pi (2a+1)m/d}-e^{-2\pi (a-x-1)m/d}]\\ & =\frac{1}{m}\cdot e^{-2\pi (a-x-1)m/d}\cdot \frac{\left(1-e^{-2\pi (x+2)m/d})(1-e^{-2\pi xm/d}\right)}{1+e^{-2\pi am/d}} \end{array}$$

After adding the row of current sources in y=1 and considering the potential contribution of the upper and lower half planes at the same time, the total potential between points 2 and 3 is

(13) $$\sum \Delta \varphi_{n}=\frac{IR_{s}}{\pi }[\pi \frac{x}{d}+\ln(1-e^{-2\pi (1+x)/d})-\ln(1-e^{-2\pi /d})+\sum_{m=1}^{\infty }a_{m}]$$

According to Ohm’s law, the resistance between points 2 and 3 can be expressed as

(14) $$R=\frac{V}{I}=\frac{R_{s}}{\pi }[\pi \frac{x}{d}+\ln(1-e^{-2\pi (1+x)/d})-\ln(1-e^{-2\pi /d})+\sum_{m=1}^{\infty }a_{m}]$$

Therefore, (14) is the theoretical model for the four-probe method to detect the resistance between two points on a finite-size conductive film.

## 3. Simulation Analysis of Resistance between Two Points on Model Surface

After the theoretical model is established in Section 2, the simulation analysis of the resistance between two points is carried out on the ITO conductive film. The ITO conductive film is produced by magnetron sputtering process [18], and the thickness and conductivity are controllable and uniform, which is suitable for quantitative analysis and repeated tests [19,20]. Therefore, ITO conductive film is selected as the research object in subsequent simulations and experiments.

**Figure 4:**
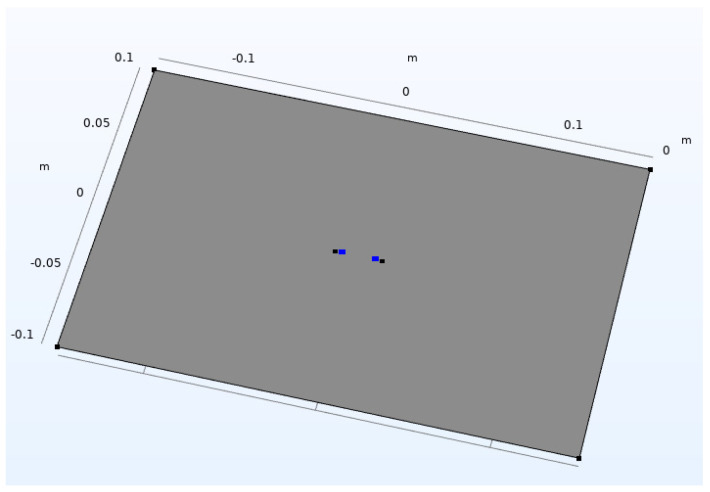
Simulation model of ITO conductive film.

**Figure 5:**
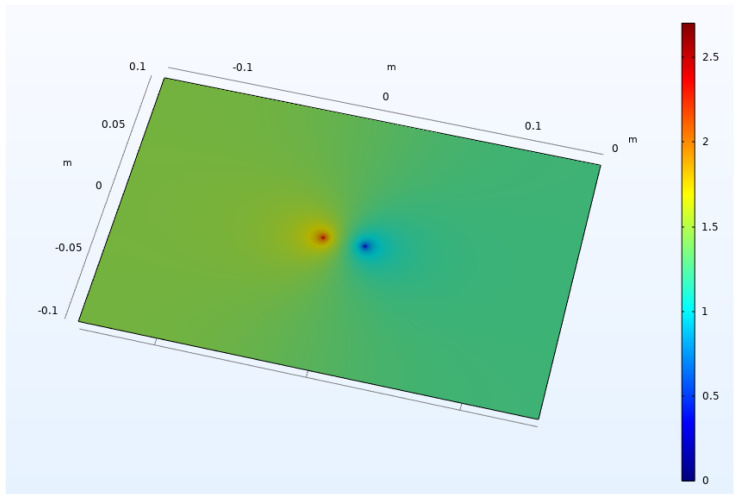
Electric field distribution of ITO conductive film (2cm between points 2 and 3).

The simulation is based on Maxwell’s equations, combined with the law of materials [21], and carried out on four ITO conductive films with different sheet resistances, as is shown in Table 1. The ITO conductive film simulation model is 0.3 m long and 0.2 m wide and shown in Figure 4. There are 4 simulation points arranged in the middle of the model, of which the leftmost simulates point 1, and the rightmost simulates point 4. Two points in the middle are used to monitor the change of potential, simulating points 2 and 3. Among them, points 1 and 2, and 3 and 4, are equally spaced; both are 4mm. The distance between points 2 and 3 is variable. The electric field distribution in the film is shown in Figure 5 as the case of 2cm between points 2 and 3. The resistance between points 2 and 3 on different films and at different spacings is obtained through electric field simulation.

**Figure 6:**
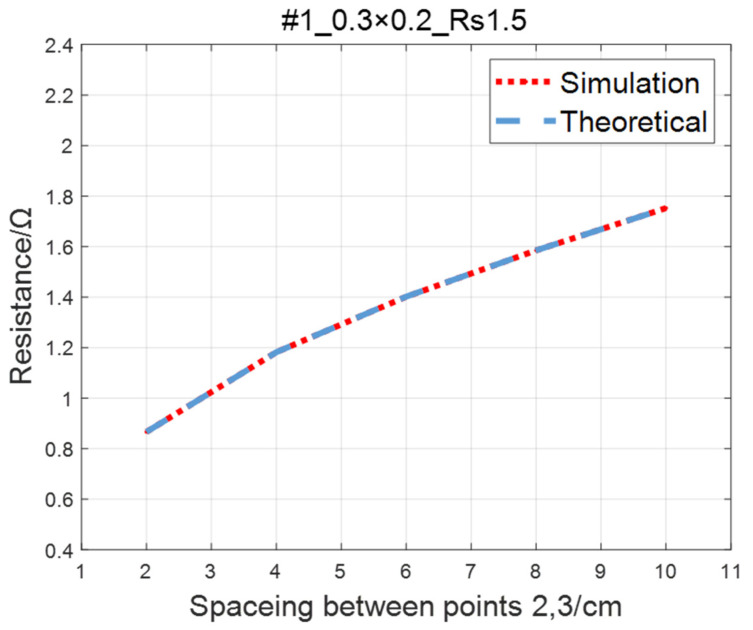
Comparison of theoretical and simulation values of #1 ITO conductive film.

**Figure 7:**
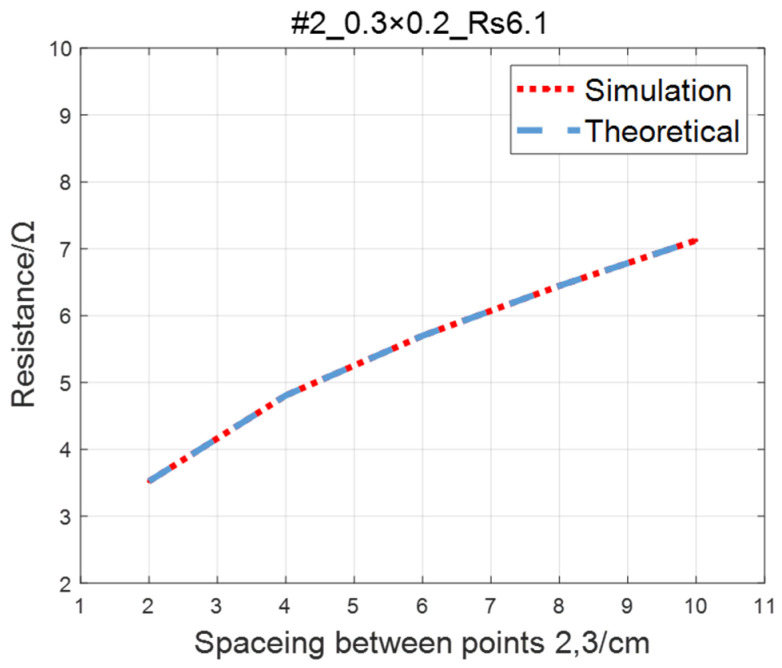
Comparison of theoretical and simulation values of #2 ITO conductive film.

**Figure 8:**
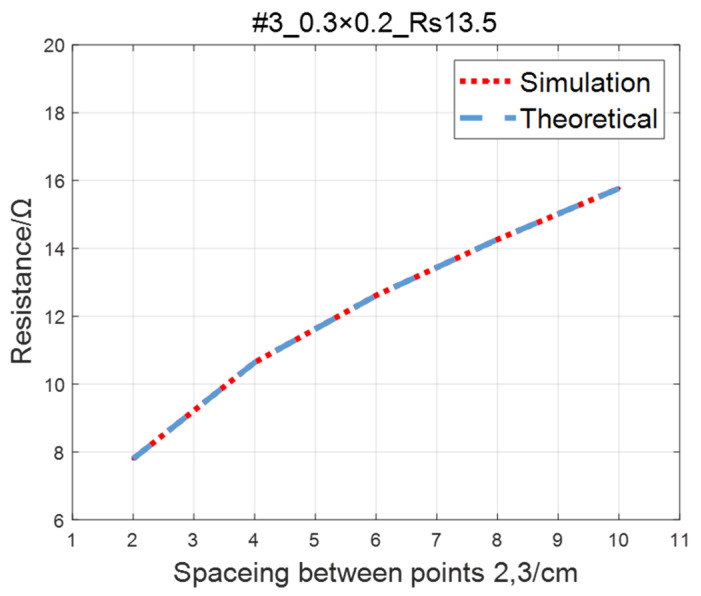
Comparison of theoretical and simulation values of #3 ITO conductive film.

**Figure 9:**
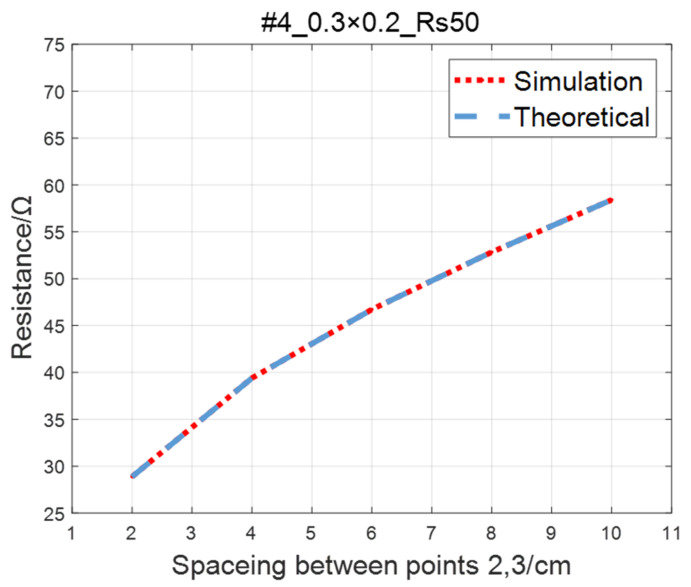
Comparison of theoretical and simulation values of #4 ITO conductive film.

Compare the simulated resistance of #1~#4 ITO conductive films with theoretical values, as shown in Figure 6, Figure 7, Figure 8 and Figure 9. The theoretical value is calculated using Equation (13). The abscissa in the figure represents the distance between points 2 and 3, and the ordinate represents the resistance between points 2 and 3. The title of the figure is divided into three parts: “#1” represents the number of the film, “0.3 × 0.2” represents the size of the film, and “Rs1.5” represents the sheet resistance. It can be seen from the figure that theoretical resistance is basically the same as simulation values. 

## 4. Experimental Verification of Four-Probe Method

After the establishment of the theoretical model of relationship between the sheet resistance and the resistance between two points on the model surface, in this part, experiments are performed on ITO conductive films with different sheet resistances to verify the accuracy of the theoretical model. The four-probe sheet resistance tester and digital micro-ohm meters are used in the experiments to measure ITO conductive films of various sizes, multiple probe pitches, and different sheet resistances.

### 4.1. Experiments on ITO Conductive Films with Dimensions of 0.3 × 0.2

**Figure 10:**
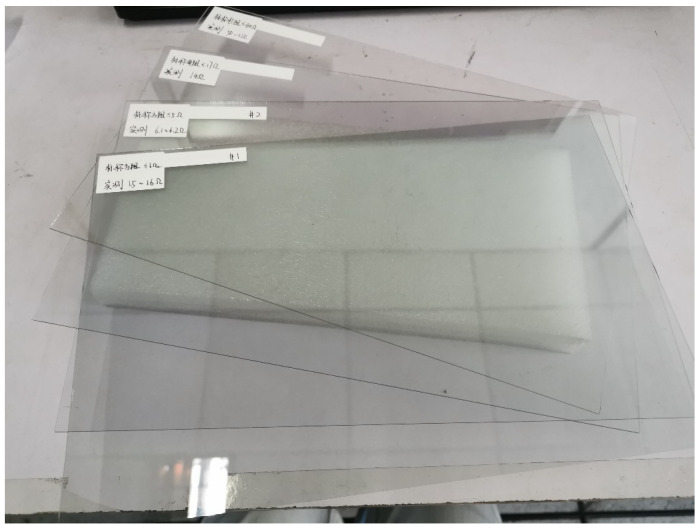
ITO conductive films.

Firstly, experiments are carried out on ITO conductive films with a length of 0.3 m and a width of 0.2 m. Four different sheet resistances are selected to test the resistance between two points with variable spacing. The film number and experimental parameters are shown in Table 2. The ITO conductive films are shown in Figure 10, with four detection points arranged side by side in the middle. The label in the figure indicates the number of ITO film and the corresponding sheet resistance, which is consistent with Table 2. The four probes of digital micro-ohm meters are, respectively, placed at four detection points, the current flows into the ITO conductive film through the leftmost probe and flows out from the rightmost probe, and the two middle probes are used for monitoring voltage. According to Ohm’s law, the ratio of the voltage to the current flowing through the conductive film is the resistance between two points in the middle.

**Figure 11:**
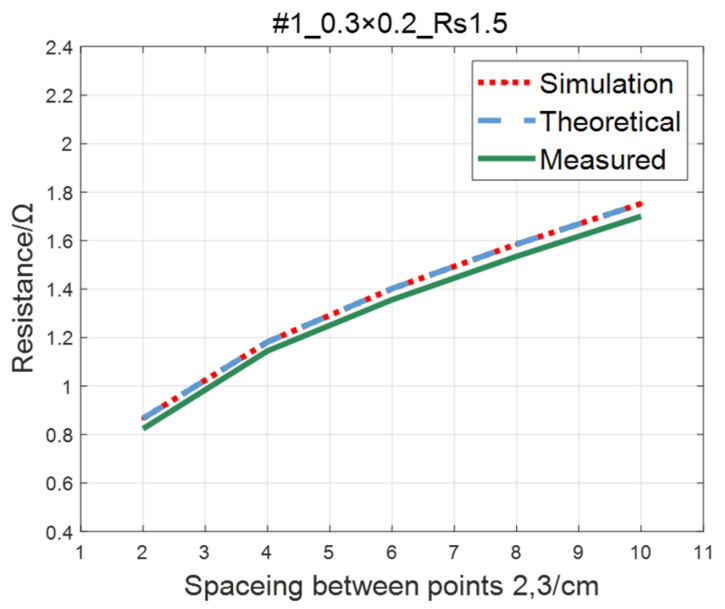
Comparison of theoretical, simulation and measured values of #1 ITO conductive film.

**Figure 12:**
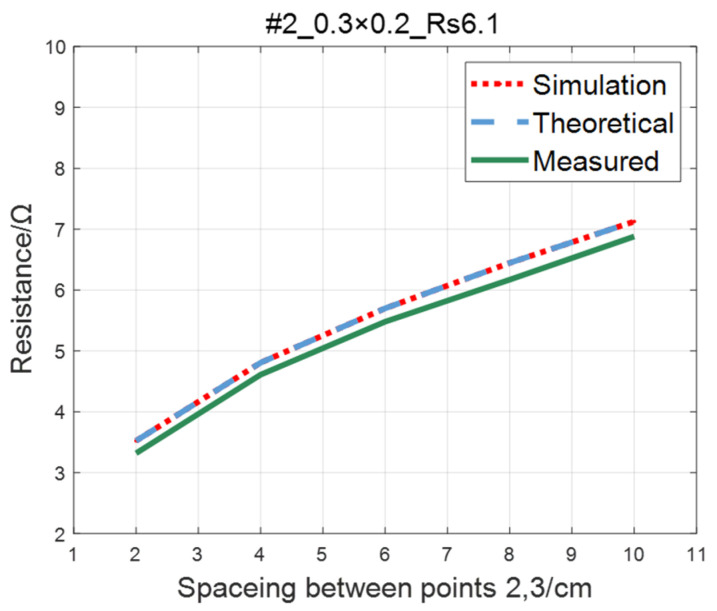
Comparison of theoretical, simulation and measured values of #2 ITO conductive film.

**Figure 13:**
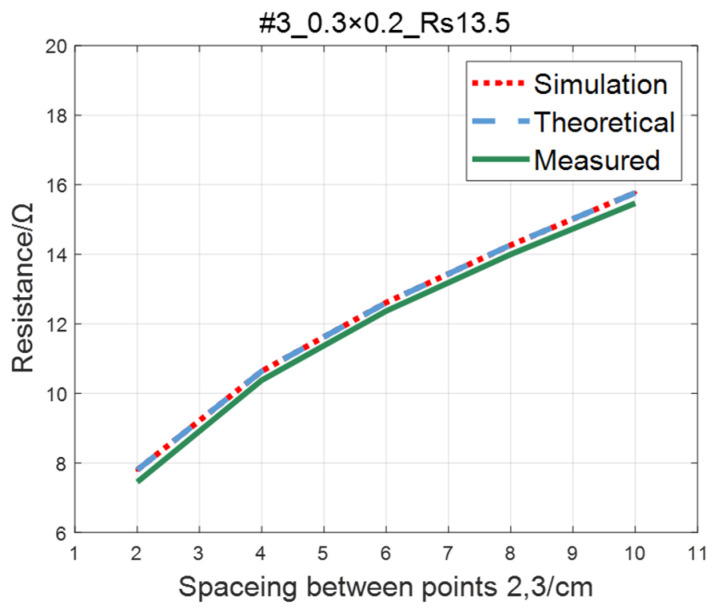
Comparison of theoretical, simulation and measured values of #3 ITO conductive film.

**Figure 14:**
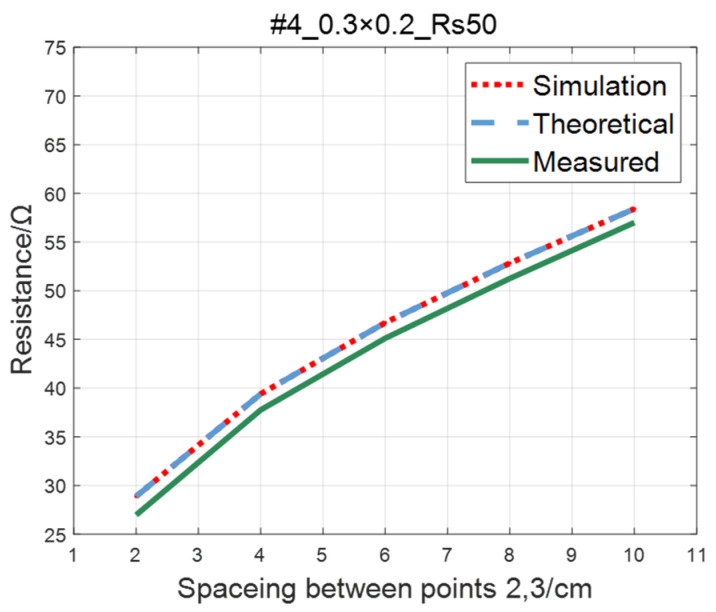
Comparison of theoretical, simulation and measured values of #4 ITO conductive film.

The comparison of the measured value, simulation value, and theoretical value of the resistance between two points on the ITO conductive film is shown in Figure 11, Figure 12, Figure 13 and Figure 14. The abscissa in the figure represents the distance between points 2 and 3, and the ordinate represents the resistance. It can be seen from the figure that the three curves have good consistency in values and trends.

### 4.2. Experiments on ITO Conductive Films with Other Dimensions

Secondly, experiments are carried out on larger ITO conductive films with a size of 0.5 m × 0.4 m, and narrow ITO conductive films with size of 0.48 m × 0.16 m, 0.48 m × 0.12 m. Randomly expand or reduce the measuring distance, among which the distance between points 2 and 3 is expanded to 300mm at maximum and reduced to 4mm at minimum. The film number, size, and sheet resistance are shown in Table 3.

**Figure 15:**
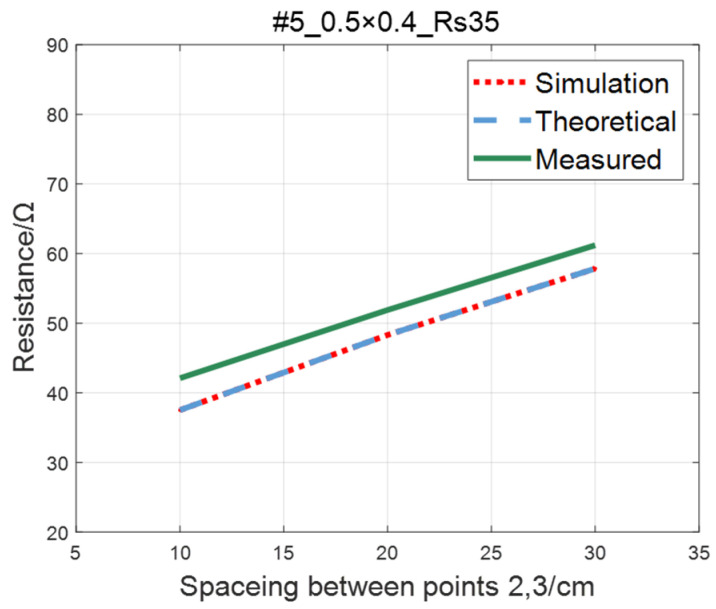
Comparison of theoretical, simulation and measured values of #5 ITO conductive film.

**Figure 16:**
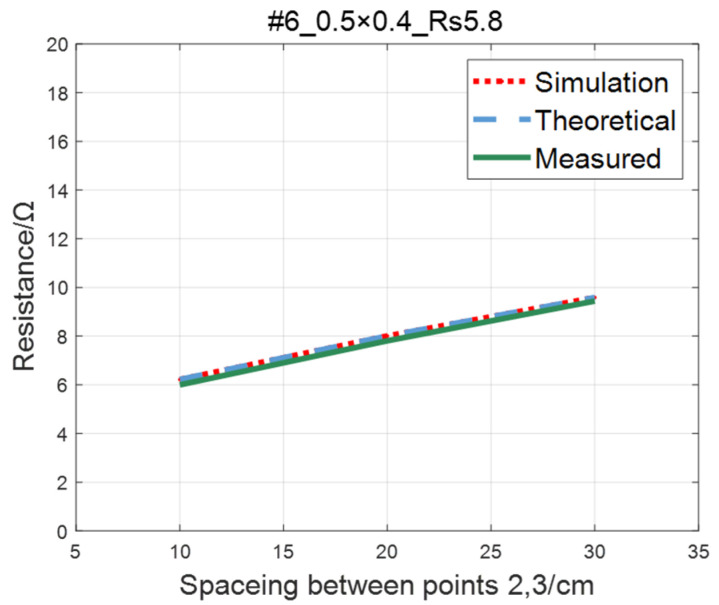
Comparison of theoretical, simulation and measured values of #6 ITO conductive film.

**Figure 17:**
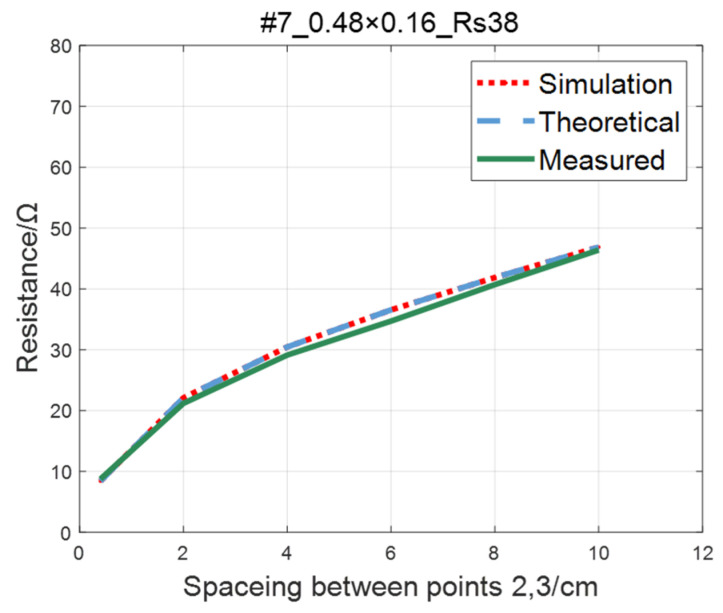
Comparison of theoretical, simulation and measured values of #7 ITO conductive film.

**Figure 18:**
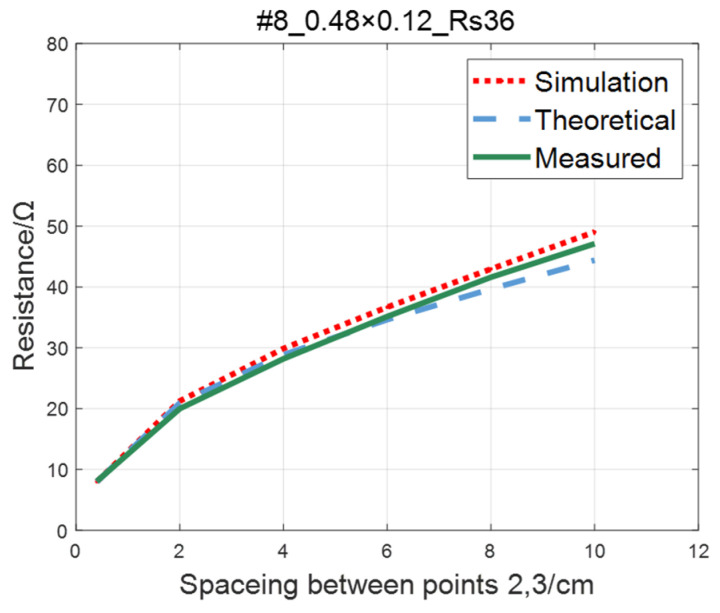
Comparison of theoretical, simulation and measured values of #8 ITO conductive film.

The comparison of the measured value, simulation value, and theoretical value of the resistance between two points on the #5–#8 ITO conductive film is shown in Figure 15, Figure 16, Figure 17 and Figure 18, where the abscissa represents the distance between points 2 and 3, and the ordinate represents the resistance. As shown in figures, the measured resistances on the #5–#7 ITO conductive films are all highly consistent with the predictions of theoretical model.

As to #8 ITO conductive film, when the spacing between points 2 and 3 is greater than 60mm, with the increase of spacing, the increasing trend of the theoretical value is significantly lower than the simulation and the measured value. This phenomenon occurs as a result of narrow width of the #8 film. The distance between points 2 and 3 gradually approaches the film width, increasing the impact of the non-conductive boundary current reflection on the potential of the detection point [22]. As a result, the simulated and measured values are slightly larger than the theoretical values.

## 5. Experimental Comparison of the Accuracy of Two-Probe Method and Four-Probe Method

Since there is no quantitative analysis on the error of the two-probe method, the following experiments are used to illustrate the measurement error of the two-probe method more intuitively.

**Figure 19:**
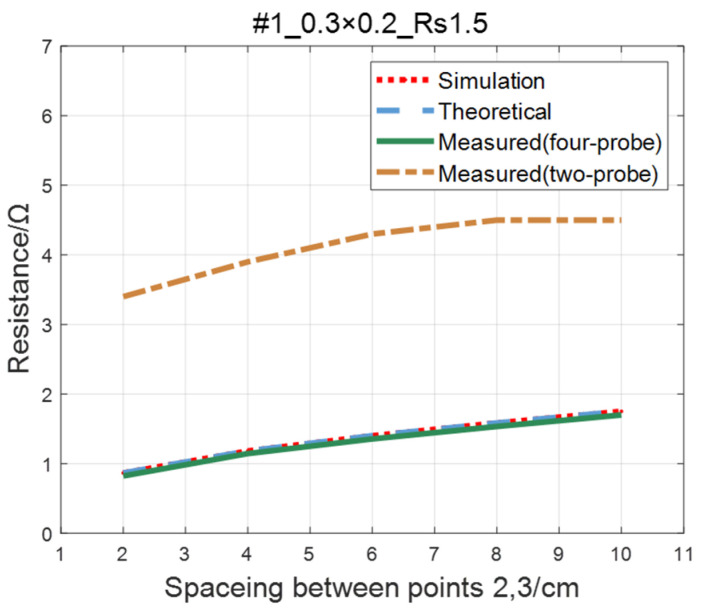
Comparison of the two-probe method and four-probe method on #1 ITO conductive film.

**Figure 20:**
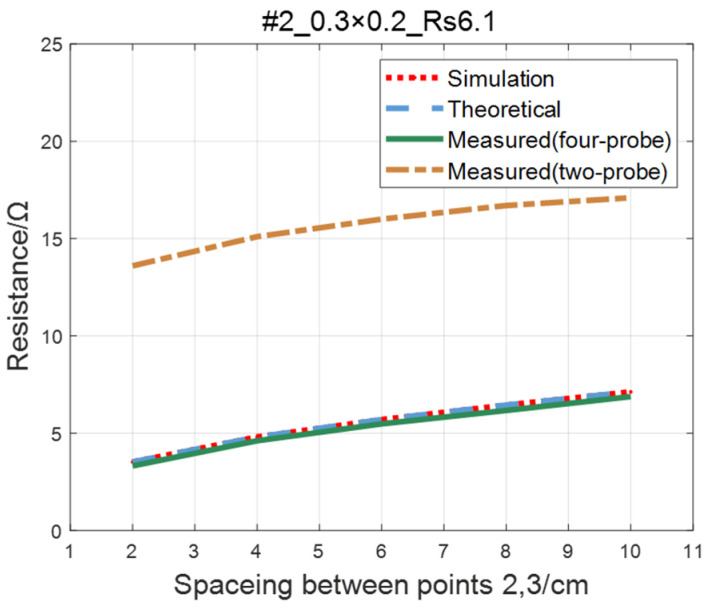
Comparison of the two-probe method and four-probe method on #2 ITO conductive film.

**Figure 21:**
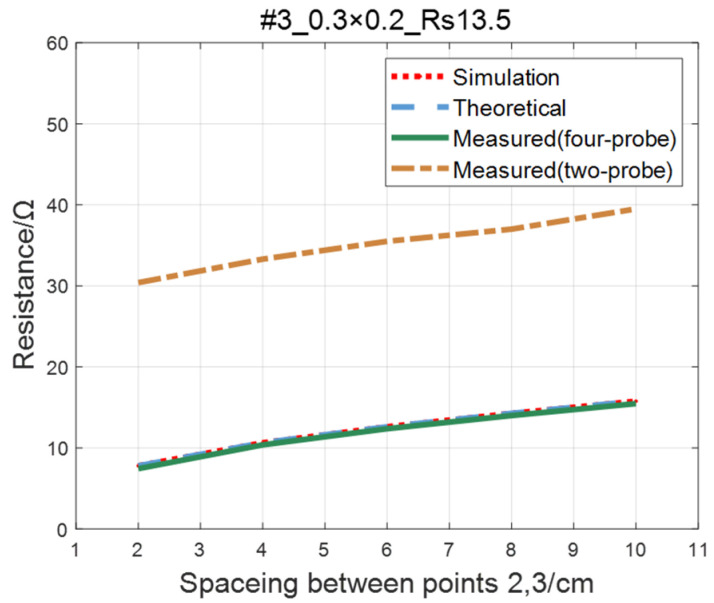
Comparison of two-probe method and four-probe method on #3 ITO conductive film.

**Figure 22:**
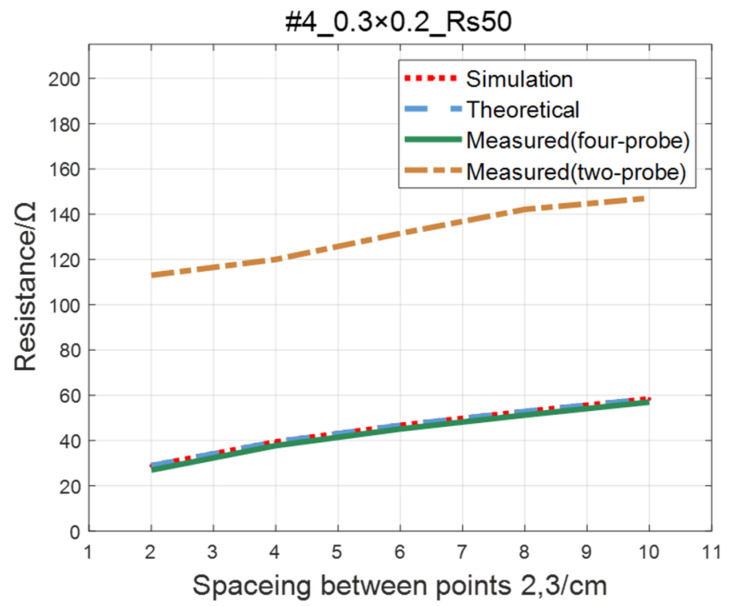
Comparison of two-probe method and four-probe method on #4 ITO conductive film.

For the ITO conductive film in Section 4.1, adopting the same experimental parameters as four-probe method (as shown in Table 2), the two-probe method is used to measure the resistance between two points with an ordinary multimeter. The measured values of the two-probe method are in contrast with theoretical, simulated, and measured values of the four-probe method, as shown in Figure 19, Figure 20, Figure 21 and Figure 22. As a result of experiments, we concluded that the measured value obtained by the two-probe method is significantly higher than the other three, with an error of 150~200%.

The application of the two-probe method will cause a greater misjudgment of the conductivity of the model surface in RCS test, thereby increasing the unnecessary spraying process and the weight of the model.

## 6. Conclusions

In order to clarify the correlation mechanism between sheet resistance and resistance between two points on the conductive film, the four-probe method is used to detect the resistance between two points on conductive films with different sheet resistances in theoretical research, simulation analysis and experimental measurement. The conclusions are as follows:

(1) The theoretical model of the relationship between sheet resistance and resistance between two points on the conductive film is proposed.
(2) The simulation method for the resistance between two points on the model surface is established.
(3) The experimental results are highly consistent with the theoretical and simulated values, which further verifies the correctness of the theoretical model and the simulation method.
(4) The measurement error of the two-probe method is significantly higher than that of the four-probe method, with an error of 150–200%.

The findings of this paper can be applicable to the following areas:

1. Fabrication of full-size/scaled model for RCS test. The method in this paper can more accurately judge whether the conductivity of the model surface sprayed with conductive paint satisfies the test requirements, thus effectively reducing the processing difficulty while maintaining the test accuracy.
2. Determine if the coating of the aircraft’s cavity (such as the cockpit) fulfills the conductivity requirements. It can prevent the electromagnetic wave from entering the cavity, thus ensuring the stealth performance of the aircraft.

With the diversity of manufacturing processes, conductive coatings are increasingly used in the aircraft design industry. There is a growing need to determine if conductive coatings match the requirements or may be substituted by simulation. The research results of this paper can continue to develop and explore greater application space.

## Figures and Tables

**Table 1:** Simulation parameters of ITO conductive films.

Film Number	Sheet Resistance	Spacing between Points 2, 3
#1	1.5 Ω/□	2 cm, 4 cm, 6 cm, 8 cm, 10 cm
#2	6.1 Ω/□	2 cm, 4 cm, 6 cm, 8 cm, 10 cm
#3	13.5 Ω/□	2 cm, 4 cm, 6 cm, 8 cm, 10 cm
#4	50 Ω/□	2 cm, 4 cm, 6 cm, 8 cm, 10 cm

**Table 2:** Experiment parameters of ITO conductive films.

Film Number	Sheet Resistance	Spacing between Points 2 and 3
#1	1.5 Ω/□	2 cm, 4 cm, 6 cm, 8 cm, 10 cm
#2	6.1 Ω/□	2 cm, 4 cm, 6 cm, 8 cm, 10 cm
#3	13.5 Ω/□	2 cm, 4 cm, 6 cm, 8 cm, 10 cm
#4	50 Ω/□	2 cm, 4 cm, 6 cm, 8 cm, 10 cm

**Table 3:** Experiment parameters of ITO conductive films.

Film Number	Size	Sheet Resistance	Spacing between Points 2,3
#5	0.5 m × 0.4 m	35 Ω/□	100 mm, 200 mm, 300 mm
#6	0.5 m ×0.4 m	5.8 Ω/□	100 mm, 200 mm, 300 mm
#7	0.48m × 0.16m	38 Ω/□	4 mm, 20 mm, 40 mm, 60 mm, 80 mm, 100 mm
#8	0.48 m × 0.12 m	36 Ω/□	4 mm, 20 mm, 40 mm, 60 mm, 80 mm, 100 mm

## Data Availability

Data is contained within the article.
